# *Streptomyces pactum* assisted phytoremediation in Zn/Pb smelter contaminated soil of Feng County and its impact on enzymatic activities

**DOI:** 10.1038/srep46087

**Published:** 2017-04-07

**Authors:** Amjad Ali, Di Guo, Amanullah Mahar, Fang Ma, Ronghua Li, Feng Shen, Ping Wang, Zengqiang Zhang

**Affiliations:** 1 College of Natural Resources and Environment, Northwest A&F University, Yangling, 712100, China; 2 Centre for Environmental Sciences, University of Sindh, Jamshoro 76080, Pakistan

## Abstract

Anthropogenic activities, such as industrial expansion, smelting, mining and agricultural practices, have intensified the discharge of potentially toxic trace elements (PTEs) into the environment, threatening human health and other organisms. To assist phytoremediation by sorghum in soil contaminated by smelters/mines in Feng County (FC), a pot experiment was performed to examine the phytoremediation potential of *Streptomyces pactum* (Act12) + biochar. The results showed that root uptake of Zn and Cd was reduced by 45 and 22%, respectively, while the uptake of Pb and Cu increased by 17 and 47%, respectively. The shoot and root dry weight and chlorophyll content improved after Act12 inoculation. β-glucosidase, alkaline phosphatase and urease activities in soil improved and antioxidant activities (POD, PAL, PPO) decreased after application of Act12 + biochar due to a reduction in stress from PTEs. BCF, TF and MEA confirmed the role of Act12 in the amelioration and translocation of PTEs. PCA analysis showed a correlation between different factors that affect the translocation of PTEs. Overall, Act12 promoted the phytoremediation of PTEs. Field experiments on Act12 + biochar may provide new insights into the rehabilitation and restoration of soils contaminated by mines.

The excessive release of potential toxic trace elements (PTEs) in the biosphere is a global concern. Rapid industrialization, agricultural activities (pesticides and fertilizers), smelting, mine exploration, coal combustion, incineration and dumping of municipal solid wastes have severely contaminated soil and water resources^1^,^2^. The toxicity of trace elements to public health, groundwater contamination, phytotoxicity, soil degradation and alteration of natural ecosystems are widely studied^3^,^4^. Trace elements can reduce the biosynthesis of chlorophyll, reduce respiration and hamper enzymatic activities in plants and adversely affect plant growth and development^5^. PTEs inhibit soil enzymatic activities as well as reduce organic matter decomposition and transformation, humus production and nutrient cycling^6^.

Soil pH, organic carbon, CEC, and Fe/Mn oxides affect the translocation and availability of PTEs to plants^6^,^7^. Soil can be polluted by smelter dust particles containing trace elements. After closure of the mines and smelting facilities, ore processing wastes persist, representing a source of toxic elements that severely damage the eco-environment^8^. This poses a risk to human and plant survival^9^. The application of pig manure to land can also add nutrients (N, P) and PTEs (Zn, Cu), which can also cause soil and water contamination. Pig manure compost (PMC) reduces elemental toxicity and transforms pig manure into a useful organic fertilizer^10^,^11^.

Biochar is a product of biomass pyrolysis and has attracted widespread attention due to its high chemical stability and PTE adsorption capacity^12^. Biochar promotes soil fertility and plant growth, alters soil microbial abundance, acts as an energy source and provides niches for soil micro-flora and fauna^13^. Biochar increases the CEC and soil organic carbon contents in soil. The higher CEC enhances nutrient retention and promotes the immobilization of organic and inorganic pollutants (PTEs)^14^. Biochar promotes the revegetation, rehabilitation and restoration of degraded lands^15^. Remediation of PTEs in soil contaminated by mines through physicochemical techniques is very expensive, laborious and unsuitable for larger mining sites. Therefore, phytoremediation assisted by biotechnological approaches in soil contaminated by smelters and mines has received great attention in recent years^16^,^17^,^18^. The remediation potential of plants is expressed as the uptake of PTEs in the shoot/root and biomass produced^19^.

Soil microbes affect the mobility and availability of PTEs through chelation, acidification and siderophore formation^20^,^21^. Rhizosphere bacteria play a vital role in phytoremediation by promoting the uptake of PTEs in plants and by increasing biomass production^22^,^23^. *Streptomyces* (Actinomycetes) are known to promote plant growth in rice, sorghum and chickpea^22^. *Streptomyces pactum* (Act12) promoted plant growth and suppressed pathogenic disease; however, its role in the phytoremediation of PTEs and soil/plant enzymatic activities has not been reported in soil contaminated by smelters/mines^24^.

Feng County is located in the southwest of Shaanxi Province (China), surrounded by the Qinling Mountains. Feng County (FC) is very rich in mineral resources. Zinc-lead mine reserves of 4.5 million tons are one of the four large bases in China. The climate in FC is dry, with a temperature ranging from −1.1 to 22.7 °C and an average annual rainfall of 613 mm. The surface soil in FC has mainly been contaminated by zinc smelters, mining, mineral processing and atmospheric deposition of waste emissions over the years^25^. This is the first scientific report on the potential role of *Streptomyces pactum* to assist phytoremediation of PTEs and promote enzymatic activities in smelter-contaminated soil and sorghum leaves. Sorghum produces large amounts of bio-mass, resists drought and promotes high uptake of PTEs in shoots^3^. The main objective of the study was to predict the potential of *Streptomyces pactum* and wood biochar in the phytoremediation of PTEs in soils polluted by smelters/mines in Feng County.

## Results and Discussion

### Characteristics of FC soil, pig manure compost and wood biochar

The main physicochemical characteristics of FC soil, pig manure compost and biochar are presented in Table 1. Soil collected from FC was neutral in nature (7.72), while the pH was neutral to alkaline (7.17 and 8.49) for the compost and biochar, respectively. The EC was reported to be 422, 5313 and 1935 μS cm^−1^ for FC soil, compost and biochar, respectively. The major soil fraction was sand (50.01%), followed by silt (48.43%) with a sandy loam texture. FC soil has a high CEC (96.5 cmol^+^ kg^−1^), which can contribute to the retention of PTEs^26^. The organic matter, total nitrogen and phosphorus were 14.9, 1.23 and 0.848 g kg^−1^ in FC soil, while they were 695.4, 25.83 and 26.5 g kg^−1^ in PMC, respectively. Biochar contained 4.51 and 0.23 g kg^−1^ total nitrogen and phosphorus, respectively. The total potassium in FC soil and PMC were 5.51 and 7.45 g kg^−1^, respectively. The total organic carbon in FC soil and PMC were 8.64 and 40.3 g kg^−1^, respectively. Adsorption/immobilization, migration and transformation of PTEs have a direct relationship with soil organic carbon and pH^7^,^12^,^27^. The presence of a small proportion of TOC in soil have prominent adsorption capacity for cations (Zn, Pb, and Cd etc.) than other minerals. TOC slow down the migration of PTEs in the soil^28^,^29^.

The total Zn, Pb, Cd and Cu contents in FC soil were 6625, 204.4, 117.7 and 51.1 mg kg^−1^, respectively. The higher Zn, Pb and Cd contents are attributed to the Zn/Pb smelting and mining activities in this area. Apart from the other PTEs, the production of recoverable Pb from mining operations in 2014 reached 2950 thousand metric tons in China^18^. The total Zn content in PMC and biochar was 1007.73 and 115.4 mg kg^−1^, respectively. PMC and biochar contained 15.7 and 25.0 mg kg^−1^ total Pb, respectively. Cd was quantified as 0.67 and 0.91 mg kg^−1^ in PMC and biochar, respectively. The total Cu in PMC was higher (675 mg kg^−1^) than in biochar (12.6 mg kg^−1^) due to the addition of Cu as an additive in pig feed^10^. The other major cations in the FC soil were Al, Fe, Ca, Na and Mg, which can possibly affect the adsorption of PTEs in a soil solution. Higher CEC favors soil aggregate stability. Divalent and trivalent cations (Ca^2+^, Al^3+^, and Fe^3+^) bridges soil clay and organic matter particles, resulting in strong aggregation^30^. Higher P and Ca can act as stabilizing agents in the immobilization of PTEs to reduce their translocation in plant tissues. Bacteria facilitate calcite precipitation in the presence of urease and Ca^31^. The total concentrations of PTEs in FC soil were in the decreasing order of Zn > Pb > Cd > Cu. The contents of trace elements were higher than the National Environmental Quality Standards for Agricultural Soil (Environmental Quality Standards, China, GB15618-1995).

The bioavailability of trace elements in soil is a dynamic process limited by chemical, biological, and environmental factors^32^. The DTPA extractable proportion of the total elemental concentration is considered to be bioavailable and is of great concern with respect to the environment and plant uptake and development^33^. The DTPA extractable concentrations of Zn, Pb, Cd and Cu were 584, 40.2, 36.4 and 1.77 mg kg^−1^ in FC soil, respectively. Feng County is a Zn/Pb mining and smelting site, which contributes to the higher bioavailable content of Zn, Pb and Cd in surface soil. The extractable Zn, Pb, Cd and Cu in PMC were 296, 5.32, 0.34 and 229 mg kg^−1^, respectively. The extractable contents of PTEs in PMC were in the decreasing order of Zn > Cu > Pb > Cd. This showed the enrichment of soil with PTEs released from PMC, which elevated the extractable concentration in the soil^34^.

### Effect of Act12 on the bioavailable fraction of PTEs

**Figure 1:**
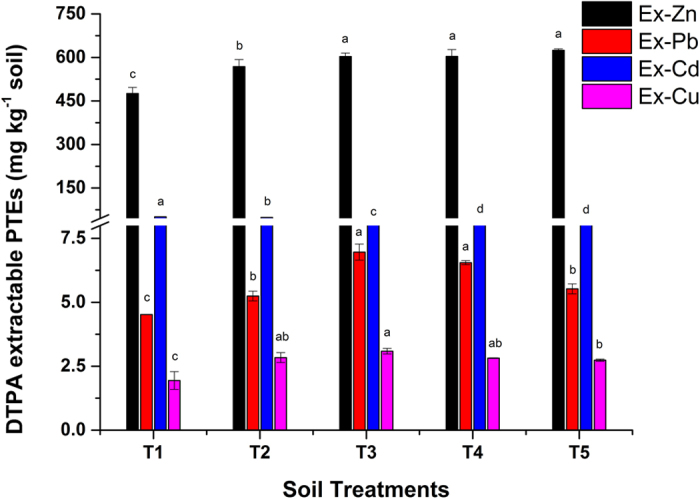
Effect of soil treatments on bioavailability of Zn, Pb, Cd and Cu in soil. Data represent the mean of three replicates and error bars are standard deviations.

The effect of soil treatments on the bioavailable fraction of PTEs is shown in Fig. 1. The elemental concentration varied after treatments. The available Zn in FC soil increased from 584 to 624.6 mg kg^−1^ (T5) due to the release of Zn from PMC into the soil solution. Act12 stimulated the degradation of organic material and facilitated the mobilization of trace elements in the soil^10^. Overall, the availability of Pb decreased to 4.52 mg kg^−1^ compared to the initial concentration in the soil (40.2 mg kg^−1^). A major portion of Pb was accumulated in the shoots and roots of sorghum.

Some Pb might also be organically bound and retained in the soil by the addition of biochar and compost, due to organic matter^26^,^29^. This implicated that the addition of biochar and Act12 favored the phytoremediation of Pb and retained it at a safe limit in soil^27^. Houben *et al*.^12^ and Chiu *et al*.^35^ also reported the diminution of Pb after the application of biochar and manure compost to mine tailings. The Cd concentration decreased from 50.51 to 36.4 mg kg^−1^ (T1–T5), but the level was almost same as reported earlier for FC soil. He *et al*.^36^ reported an increase in the Cd fraction following inoculation by *Pseudomonas sp*. RJ10. The Cu concentration was almost as high in all treatments after the soil treatments. The higher Cu content is attributed to the 5% Cu-rich PMC applied as a nutritional supplement to each pot. PMC has high Zn and Cu contents, which are released after soil treatments. This could be the possible reason for the higher contents of Zn and Cu in the soil after the sorghum harvest^11^. Overall, the concentrations of Pb, Cd and Cu decreased in T3–T5 possibly because of urease and microbial-induced calcite precipitation, as well as biochar-induced immobilization of PTEs^12^, which reduced the uptake and availability of PTEs in contaminated soil^31^.

### Effect of Act12 on the accumulation of PTEs in sorghum biomass

The role of Act12+ biochar in the shoots and roots of sorghum was assessed in terms of the uptake of PTEs. Act12 and biochar increased the uptake in shoots and roots of sorghum grown on FC soil polluted by mines. Adsorption and precipitation of PTEs on compost and microbial cells might be the possible reasons for the decrease in uptake^31^,^37^.

### Accumulation of PTEs in sorghum shoots

**Figure 2:**
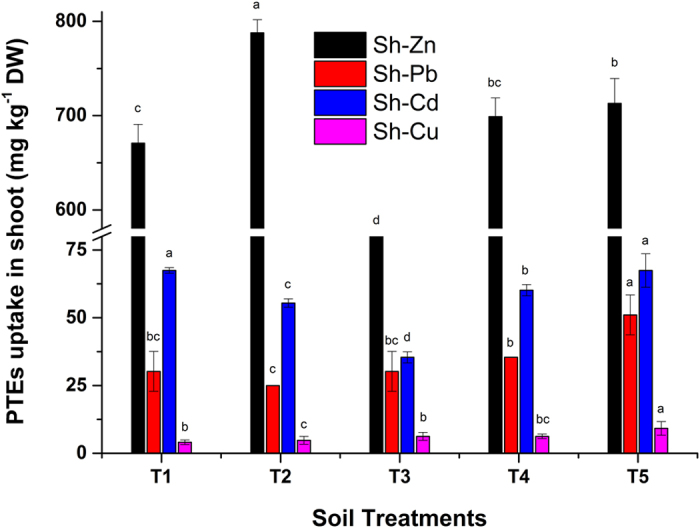
Effect of soil treatments on shoot uptake of Zn, Pb, Cd and Cu in *Sorghum bicolor*. Data represent the mean of three replicates and error bars are standard deviations.

The uptake of PTEs (Zn, Pb, Cd and Cu) in sorghum shoots showed a positive relationship with the extractable content of DTPA in FC soil. The effect of 1% biochar addition with different levels of Act12 was quantified in terms of the translocation of PTEs in sorghum shoots and roots. However, the distribution of individual trace elements in sorghum shoots (Fig. 2) differed in each treatment. Zn accumulation in shoots was significantly influenced by the additions, and Zn accumulation in sorghum shoots showed a 17% increase in T2 compared to the control (T1). Zn uptake in sorghum shoots increased in all treatments with Act12 applications, except in T3 (1% biochar + 0.075% Act12). The increase in uptake could be due to higher biomass production. Act12 enhanced root and shoot development, which favored Zn uptake in sorghum shoots^21^. Similarly, Dary *et al*.^17^ and Sheng *et al*.^16^ also reported an increase in Zn, Pb and Cd uptake due to high biomass production only after inoculation of seed/soil with plant growth-promoting microbes.

Pb uptake in sorghum shoots grown in FC soil increased in all treatments, except for T2 (1% biochar), due to the adsorption on the surface of the biochar^27^. Biochar improves the CEC and TOC of soil, which can have important effects on binding cations/anions in the soil to increase nutrient retention and availability to microbes and plants^12^,^26^. The maximum increase in uptake was reported in T5 (69%) compared to the control. Similarly, *Pseudomonas aeruginosa* increased the bioavailable Pb fraction by the production of siderophores in the rhizosphere, hence increasing uptake in maize^20^. These results are supported by the previous findings of Dary *et al*.^17^.

Cd accumulation in sorghum shoots significantly decreased. Cd accumulation in shoots harvested from FC soil decreased in T2 (18%), T3 (48%) and T4 (11%) and remained constant in T5. There was a negative relationship between the Cd uptake by sorghum and Act12 application in FC soil. Earlier studies also reported a decrease in Cd uptake and an increase in Pb uptake in *Trifolium* and *Salix caprea* after inoculation with *Brevibacillus* and *Streptomyces AR36*, respectively^1^. The variation in uptake behavior is influenced by the production of siderophores by Act12 and its erratic activity in different conditions^20^. Sinha and Mukherjee^38^ also reported a 36.89% reduction in Cd uptake in shoots of *Cucurbita pepo* and *Brassica juncea* after inoculation with *Pseudomonas aeruginosa*. Another explanation could possibly be microbial (Act12) and biochar-assisted immobilization or stabilization of Cd, and higher dose applications facilitated the uptake^31^. The addition of biochar increased the soil pH and TOC, which influenced the distribution and transformation of PTEs in soil^7^,^29^. The extractable Pb content is higher than that of Cd, but Cd uptake in sorghum shoots is greater than that of Pb. The higher Cd uptake is attributed to plant selection for the transportation of specific elements into shoots, as well as soil physicochemical characteristics^39^.

Cu showed a linear increase in uptake in sorghum shoots. A maximum increase of 125% in shoot uptake was recorded in T5, followed by 53% in T3 and T4. The higher uptake in shoots is attributed to the release of Cu from PMC by the activity of Act12^10^, as shown in Fig. 1. Elouear *et al*.^34^ also reported an increase in the uptake of trace elements in the aerial parts of alfalfa after the application of sheep manure. The uptake of Cu in shoots is low compared to the other elements because it is the least available fraction in soil^40^.

### Accumulation of PTEs in sorghum roots

**Figure 3:**
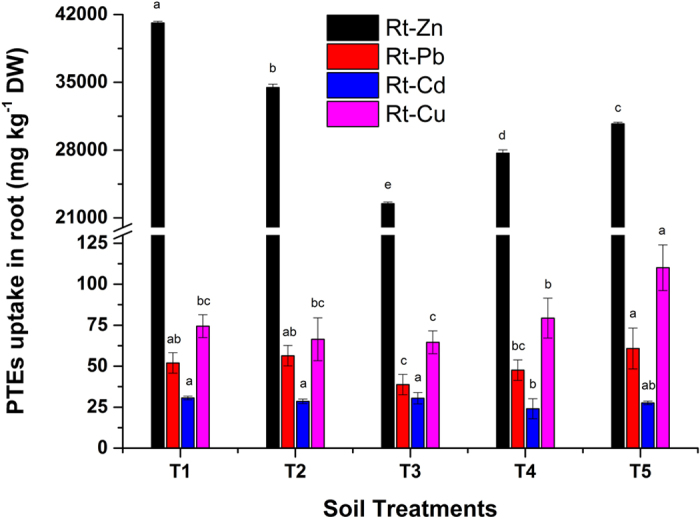
Effect of soil treatments on root uptake of Zn, Pb, Cd and Cu in *Sorghum bicolor*. Data represent the mean of three replicates and error bars are standard deviations.

Biochar application in combination with Act12 significantly influenced the uptake of PTEs in sorghum roots (Fig. 3). As far as Zn uptake in roots is concerned, no increase was reported. The decrease in uptake of PTEs is due to the stabilization (biosorption) and immobilization (precipitation) effect of the biochar and Act12 cells, respectively^41^. Previous scientific reports also revealed that microbial biosorption, accumulation and immobilization of trace elements could be appropriate mechanisms for phytostabilization in polluted soils^31^,^42^.

Pb uptake in roots showed 8 and 17% increments in T2 and T5 and 25 and 8% decreases in T3 and T4, respectively, compared to T1. Biochar facilitated the adsorption (immobilization) of Pb in the soil solution, hence causing Pb translocation into sorghum roots^12^,^27^. Yang *et al*.^18^ also reported increases in the uptake of Pb in the roots of different legumes in Pb-contaminated soil. Soil microbes favor the PTEs immobilization by the excretion of organic substances and viscous slime outside the bacterial cell. Similarly, diminution in Pb, Ni and Cd uptake was also reported in *Phaseolus vulgaris, pine, and Solanum lycopersicum*^43^,^44^.

The bioavailable fraction of Cd was higher (36.4 mg kg^−1^) in FC and indexed as highly Cd-polluted soil due to mining operations^25^. Data revealed the adverse effects of Cd in roots. Highly contaminated FC soil showed lower Cd uptake in sorghum roots. Sorghum was capable of tolerating the stress from PTEs after the application of biochar and Act12 in highly Cd-contaminated FC soil. Biochar and Act12 applications reduced the available Cd from 50.52 to 36.4 mg kg^−1^ in FC soil (Fig. 1) due to the higher adsorption capacity of biochar and microbial cells^41^,^42^. The reduction is believed to be due to increased urease formation, soil CEC and organic carbon. Urease promotes the formation of insoluble precipitates in the presence of urea and calcium^31^, while biochar assisted the increase in soil pH, which can help form CdCO_3_^45^. Higher CEC at high pH favors the formation of stable soil aggregates of clay and organic matter in the presence of cations i.e. Zn, Pb, Al, Fe etc^30^. Sinha and Mukherjee^38^ also reported a 47.70% reduction in Cd uptake in roots of *Cucurbita pepo* after inoculation with *Pseudomonas aeruginosa*.

Highly Cu-rich PMC is responsible for augmenting the bioavailable concentration^11^ in soil (3.09 mg kg^−1^) compared to the initial concentration in soil (1.77 mg kg^−1^). Cu is added to the feed of pigs and can be a possible environmental pollutant after excretion^10^. The application of wood biochar resulted in a slight decrease (10.8–13.22%) in Cu in roots collected from T2 and T3. The Cu concentration in sorghum roots increased in T5 (48%) compared to the control. Mahar *et al*.^25^ also reported a 71% increase in root Cu uptake in Chinese cabbage grown on mine soil.

### Plant growth-promoting features of Act12

The effect of Act12 and biochar on the shoot and root dry weights and chlorophyll content in sorghum grown on smelter-contaminated FC soil were used as indicators of plant growth promotion.

### Promotion of shoot and root dry biomass

**Figure 4:**
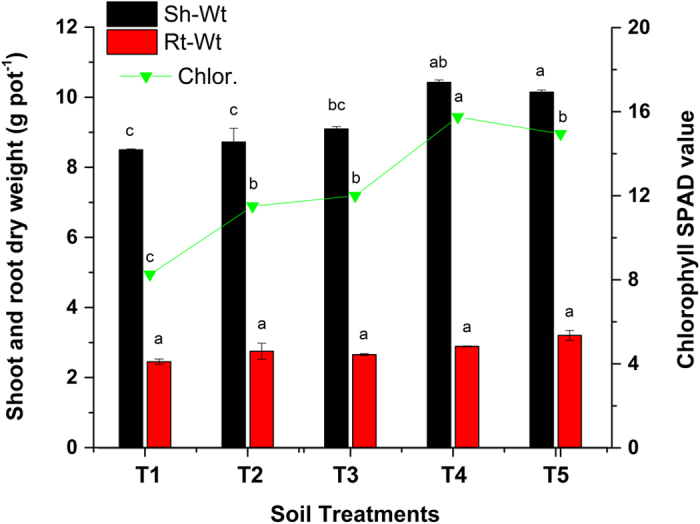
Effect of soil treatments on physiology of *Sorghum bicolor* in polluted soil. Data represent the mean of three replicates and error bars are standard deviations.

The positive effects of Act12 and biochar on shoot and root dry biomass is shown in Fig. 4. The results showed the adverse effect of PTEs on shoot and root development in smelter-contaminated FC soil. A maximum increase of 23% in shoot dry biomass was reported in T4 compared to the control (T1). Comparatively low shoot weights in FC soil are attributed to high contamination from PTEs caused by smelting, mineral processing and atmospheric deposition over the years in Feng County^25^, which inhibited plant growth and soil microbial activity. Houben *et al*.^12^ observed comparatively higher biomass yields after biochar application, whereas toxic elements (Zn, Pb and Cd) were low in soil. Gopalakrishnan *et al*.^22^ also reported an increase in root/shoot biomass of chickpea after inoculation with *Streptomyces*. Al Chami *et al*.^3^ reported 58 and 82% (shoot) and 67 and 91% (root) diminution in *Sorghum bicolor* after applications of 100 mg kg^−1^ Zn and Pb, respectively. Shoot development is usually enhanced by enzymatic activities, siderophore and indole acetic acid production by the *Streptomyces*^21^. Siderophores form organic complexes with trace elements (Zn, Cu, Cd, Al and Pb) to alleviate stress on plants^46^.

Toxic trace elements retard cell division and elongation. Rhizosphere microbes work symbiotically with plants and have the potential to detoxify hazardous waste in roots^47^. The results showed a maximum increase of 27% in root dry weight in T5 (1% biochar + 0.225% Act12). PTEs inhibited plant growth in control pots (T1) due to stress, whereas Act12 and biochar reduced the stress by producing antioxidant enzymes and adsorbing PTEs, respectively. Antioxidant enzymes inhibit ethylene production and thus improve the shoot/root development^48^. Poor root growth weakens a plant’s ability to take up nutrients and water. Shoot and root development is highly influenced in FC soil due to the high toxicity of Zn, Pb and Cd. Similar findings were also reported by Manquián-Cerda *et al*.^49^. Other scientific reports also revealed 4–25% (root) and 2–55% (shoot) increases in sorghum and rice, respectively, after inoculation with various strains of *Streptomyces*^21^,^22^.

### Chlorophyll assessment

Biochar and Act12 significantly increased the chlorophyll content in sorghum leaves (Fig. 4). The maximum increase in the chlorophyll content was reported in T4 (91%) compared to the control. Feng County is mainly polluted by Zn, Pb and Cd (Table 1). In this study, visual observations of chlorotic spots on sorghum leaves were observed due to the higher total Zn content (6625 mg kg^−1^) in FC soil. Lead can cause physiological and biochemical dysfunction in plant growth, nitrate assimilation and photosynthesis^6^. Cd is known to inhibit chlorophyll biosynthesis in plant leaves^2^. Manivasagaperumal *et al*.^4^ reported a decrease in plant shoot and root growth, antioxidant enzymatic activity and chlorophyll content due to contamination with PTEs. The addition of biochar and Act12 reduced the stress from PTEs and improved the chlorophyll content in each treatment. Leaf chlorophyll is reduced by ROS stimulation mediated PTEs and lipid peroxidation. Siderophores-producing bacteria have been reported to enhance the chlorophyll content^50^. The introduction of *Pseudomonas aeruginosa* KUCd1 and *Enterobacter aerogenes* NBRI also enhanced the chlorophyll content, shoot/root length and dry weight in *Cucurbita pepo* and *Brassica juncea*, respectively^47^. Our results confirmed the role of biochar and Act12 in enhancing the shoot/root growth and chlorophyll in sorghum grown on a smelter-contaminated FC soil.

### Effect of Act12 on soil enzymatic activity

**Figure 5:**
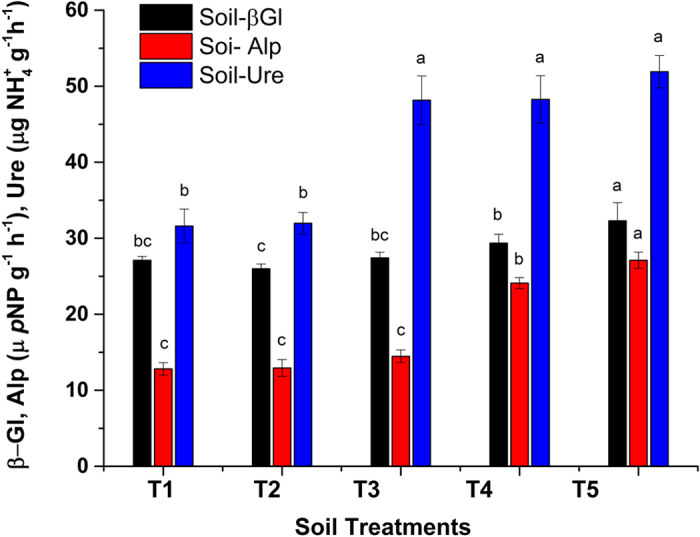
Effect of soil treatments on enzymatic activities in smelter/mines polluted soil. Data represent the mean of three replicates and error bars are standard deviations.

Soil enzymes act as biological catalysts to facilitate biochemical reactions, organic matter decomposition and transformation; detoxify pollutants; produce essential compounds for microbes and plants; synthesize humus; and maintain soil structure^6^,^42^. β-glucosidase, alkaline phosphatase and urease were found in FC soil (Fig. 5). PTEs interact with the enzyme–substrate complex, denature the enzyme or interact with its active groups, as well as affect the synthesis of enzymes by microbial cells, which affects the enzymatic activity in soil^33^. Higher concentrations of PTEs result in a lower enzymatic activity, which reflects the pollution severity in soil. Enzyme-clay and enzyme-organic polymer complexes resist denaturation^6^.

β-glucosidase releases low molecular weight sugars that act as energy sources for soil microbes^51^. β-glucosidase was significantly enhanced after treatments. β-glucosidase showed a 19.10% increase in T5 (1% biochar + 0.225% Act12). Biochar application in T2 showed a decrease in β-glucosidase due to adsorption on the surface of the biochar, which reduces the amount of substrate for enzymatic activity^27^,^52^. Co-application of Act12 and biochar enhanced microbial activity, and the β-glucosidase content was reportedly high in T3–T5. Although FC soil is more polluted due to smelting and mining processes, soil samples were collected from a farmer’s maize field near the smelter and 5% PMC was added. Cultivated lands are rich in organic matter (N, P), which might be a possible reason for the higher β-glucosidase activity in FC soil^53^.

Alkaline phosphatase activity was improved after application of biochar and Act12. PTEs are responsible for adverse effects on alkaline phosphatase activity in polluted soils^5^. A maximum increase of 111.5% in alkaline phosphatase activity was observed in T5 (1% biochar + 0.225% Act12) due to the substrate enrichment in FC soil. The soil P content is optimum for plant growth and enzymatic activity (Table 1). Furthermore, the addition of 5% PMC along with 1% wood biochar added to each pot with different levels of Act12 (T3–T5) improved the phosphatase activity due to phosphorus solubility^26^. The stress from PTEs was reduced after the biochar and Act12 treatments in the studied soils due to adsorption and phytoremediation, respectively^42^.

Urease can lead to calcite formation by hydrolyzing urea and increasing the pH to accelerate chemical reactions. The pH disturbance can affect the translocation of PTEs and availability in plants, roots and soil^31^. Urease is responsible for the transformation of plant nutrients and soil organic matter. Urease activity was enhanced by biochar and Act12 and showed a 64.27% increase in T5 (1% biochar + 0.225% Act12). The higher urease activity is attributed to the higher nitrogen content in PMC, which is released into soil^54^. Overall, the soil enzymatic activity decreased in the order of urease > β-glucosidase > alkaline phosphatase. This could be due to the higher contents of nitrogen, organic matter and phosphorus in PMC. Therefore, stress from PTEs was reduced by the application of wood biochar and PMC, and the overall enzymatic activity was promoted^33^. Our results confirmed that the presence of PTEs released from smelter emissions significantly affected the enzymatic activity in soil in FC. The addition of biochar and Act12 improved the soil ecosystem quality and functional diversity and helped cope with pollution from PTEs^5^.

### Effect of Act12 on plant enzymatic activity

Abiotic stresses, such as PTE contamination, drought, humidity and salinity, potentially increase ROS formation, which damage plants by oxidizing pigments, proteins, membrane lipids, and amino acids. Plants cope with ROS using antioxidant enzymes (POD, PAL and PPO) and lipid peroxidation (MDA). Under stressful conditions, a high antioxidant capacity prevents damage resulting from ROS formation^2^. Thus, the activity of antioxidant enzymes is frequently used as an indicator of oxidative stress in plants grown on soil contaminated by PTEs^55^.

**Figure 6:**
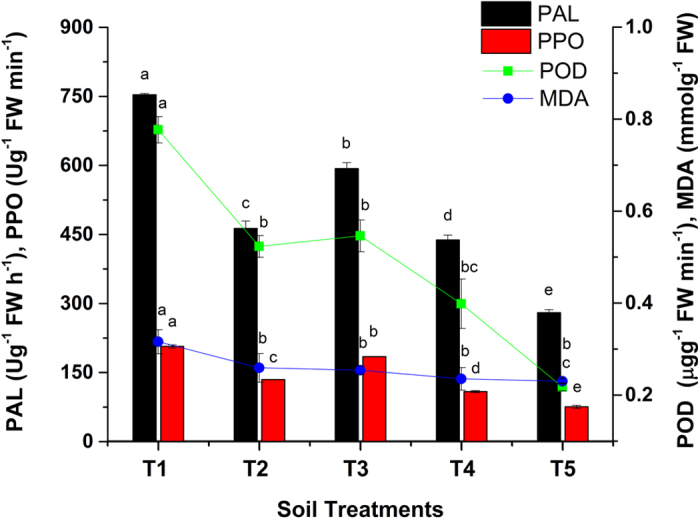
Effect of soil treatments on antioxidant enzymatic activities and MDA content in sorghum leaves. Data represent the mean of three replicates and error bars are standard deviations.

POD is a plant-specific oxidoreductase with multiple catalytic activities, and its activity changes in response to stress posed by PTEs. POD is involved in photosynthesis, protein metabolism, regulation of plant growth, reduction of Cu toxicity and detoxification of free radicals^40^,^56^. The POD results showed that the activity of antioxidants decreased in sorghum after application of Act12 in soil at rates of 0.075, 0.15 and 0.225% (Fig. 6). Stress from PTEs was high in T1 plants, and after Act12 application, a 72% decrease in POD occurred. This demonstrated the effectiveness of biochar and Act12 in reducing oxidative stress in sorghum. The collected soil from FC was mainly polluted by Zn, Pb and Cd. POD activity significantly decreased with increasing doses of Act12 and biochar due to enhanced plant growth and adsorption of PTEs, respectively. Similarly, POD activity decreased linearly with diminishing stress from PTEs in *Glycine max* and *Vicia faba*^57^. POD activation might be due to the high Zn content and exceeding the threshold level of Cd (10 mg kg^−1^) in smelter-contaminated FC soil^19^. These findings are in agreement with Siddiqui and Meon^58^, who observed a reduction (30%) in POD after inoculation of *Capsicum annuum* seeds with *Pseudomonas aeruginosa*.

PAL activities decreased by 63% (T5) in sorghum leaves (Fig. 6). Data showed comparatively high levels of PAL in sorghum due to the high contamination of soil with PTEs (Zn, Pb and Cd). Biochar alone is more effective than Act12, but a combination of both is highly effective in reducing oxidative stress. Seed inoculation with symbiotic microbes helps plants survive in the presence of PTEs by promoting antioxidant activity^23^. Higher peroxidase activity in bacteria-inoculated *Ricinus communis* and *Helianthus annuus* grown on polluted soil has also been reported^59^.

PPO oxidizes phenols to chinone. POD activity is mainly affected by the Zn content^60^. The activity of PPO is higher compared to that of POD and MDA in highly Zn-loaded FC soil (Fig. 6). Similarly, the PPO activity in sorghum leaf tissues was also significantly reduced by increasing the biochar + Act12 doses. The maximum reduction (63.5%) of the PPO level was reported in T5 (1% biochar + 0.225% Act12). Sorghum can sense low concentrations of PTEs (Zn, Pb and Cd) as potential oxidative stress and stimulates antioxidant activity, which results in lowering the amount of ROS and reducing oxidative damage^19^.

### Lipid peroxidation products

Malondialdehyde (MDA) is a product of lipid peroxidation caused by oxidative damage in sorghum leaves. The effect of wood biochar and Act12 on the MDA content in sorghum leaves compared to the control is shown in Fig. 6. The MDA content decreased by 27.2% in sorghum leaves. The results showed that biochar alone and in combination with Act12 linearly reduced the stress of PTEs on sorghum grown on soil contaminated by mines as well as increased resistance to membrane damage^61^. Previous scientific reports have revealed a direct relationship between increasing MDA, POD and PAL levels with rising concentrations of Cd, As and Cu, respectively^2^,^48^. Hence, lipid peroxidation is amplified by stress from PTEs in plants. Oxidative stress induced by PTEs initially increased the MDA content, followed by increases in antioxidant enzymatic activities (POD, PAL and PPO) and, finally, degraded chlorophyll^49^. Our findings showed that the application of Act12 and biochar enabled sorghum to produce antioxidants to protect it from oxidative damage caused by PTEs in soils polluted by smelters and mines^40^.

### Phytoextraction indices of PTEs

The phytoextraction efficiency of sorghum assisted by Act12 grown in soil contaminated by the mines and smelter in FC was assessed by BCF, TF and MEA, as shown in Table 2. The BCF values of Zn, Pb, Cd and Cu in FC shoot samples increased to a maximum of 1.34, 1.27, 1.85 and 5.19, respectively, after Act12 inoculation, which is greater than the critical value (1.0) for a hyperaccumulator. The higher BCF values show the potential role of Act12 in the uptake of PTEs in sorghum shoots. The TF values of PTEs (Zn, Pb and Cu) were lower than the critical values (>1.0) for an ideal hyperaccumulator in the case of sorghum samples. However, the TF value for Cd was almost as high in all treatments. This confirmed that sorghum is a better hyperaccumulator for Cd translocation in soil contaminated by mines. A previous report showed that Act12 can improve Cd translocation in spiked soil^62^. However, our results from this experiment showed that the efficiency of Act12 decreases in FC soil contaminated by multiple PTEs. The lowest TF of Zn could be caused by the saturation of sorghum plants with Zn, and no further translocation can be achieved by inoculation of Act12. MEA denotes the quantity of PTEs translocated within the plant biomass when grown on a polluted substrate. The MEA results showed that Zn extraction reached a maximum of 1457 μg plant^−1^ in T4 (1.45 g plant^−1^), which was far greater in FC sorghum biomass compared to the other PTEs. The MEA value for Cd was also high in FC with higher Act12 inoculation (136.8 μg plant^−1^, T5). The MEA values for Pb were also almost as high (103.6 μg plant^−1^) in FC compared to Cu (18.66 μg plant^−1^) in T5. The MEA value of Cu was comparatively much lower than Zn, Pb and Cd. The MEA results confirmed that Act12 promoted the uptake of PTEs in FC soil and revealed that sorghum inoculated with Act12 + biochar can remediate polluted sites due to higher biomass production and the translocation of PTEs, which further illustrates that multiple cropping systems can be a better option to remediate soils contaminated by mines. Similar results were also reported by other researchers after inoculation with bacteria in polluted soil^63^.

### Multivariate statistical analysis

**Figure 7:**
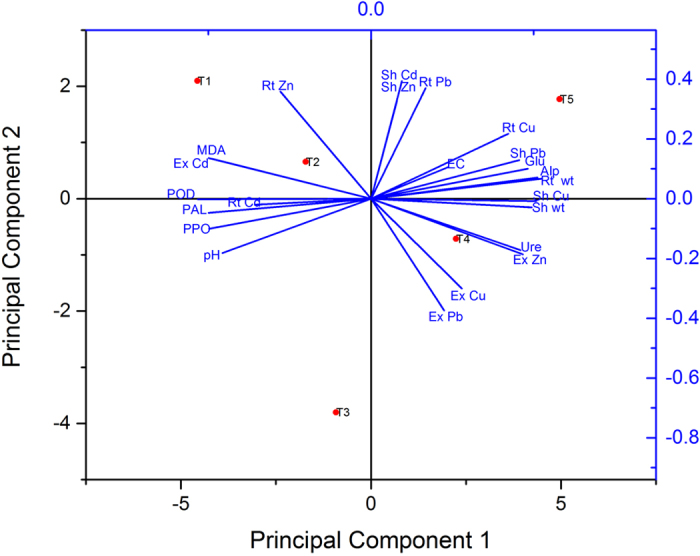
PCA analysis of different factors affecting PTEs translocation in *Sorghum bicolor*. Elements with Sh represents the shoot elements, Rt represents the root elements, POD, PAL, PPO are plant antioxidants, while Glu, Alp and Ure are soil enzymes.

Principal component analysis (PCA) is a data reduction procedure (metals concentration, other parameters of the soil or plants) applied to model large-scale data and can provide an easy visualization of latent relationships (principal components) among variables in large and complex datasets^64^. PCA was performed to draw a relationship between different factors (metal uptake, enzymatic activity of soil and plant) that can assist the translocation of PTEs and their effect on sorghum grown in mining sites^30^. The translocation of PTEs in sorghum depends on the agrochemical characteristics of soil, that is, the soil pH, EC and clay content^65^. The PCA results for all experimental parameters are shown in Fig. 7. Soil extractable Cd and MDA showed a very close correlation that affected plant growth and development. Cd can cause higher production of ROS and increase the MDA content, which can eventually lead to plant death^49^. The Cu content in the soil was low, and higher shoot biomass and soil EC facilitated Cu translocation in the sorghum shoots. As mentioned, Cu ions are released from the PMC and translocated into the sorghum shoots. Plant enzymes, such as POD, PAL and PPO, were also influenced by the soil pH and Cd translocation in roots. The plant enzymatic content linearly decreased with the concentration of Cd in soil, showing that stress was reduced after the uptake of Cd by sorghum^57^.

## Conclusions

Different levels of *Streptomyces pactum* with 1% biochar were used to enhance sorghum growth in soil polluted by mining in Feng County, Shaanxi Province, China. The bioavailable fraction of Zn and Pb increased in soil from release from the PMC, and Cd remained stable, while Pb was successfully reduced after treatments due to adsorption on the surface of the biochar. Zn, Pb and Cu uptake in sorghum shoots significantly increased and Cd uptake declined in shoot samples after soil treatments. However, Zn and Cd uptake in roots decreased, while Pb and Cu uptake increased after Act12 + biochar application. Plant growth-promoting features (shoot and root dry weights and chlorophyll) significantly increased in Act12 inoculated pots. The soil enzymatic activity (β-glucosidase, alkaline phosphatase, urease) was improved. Antioxidant enzymatic activity, such as POD, PAL, PPO and lipid peroxidation (MDA), significantly decreased in sorghum leaves. The decrease plant antioxidant activity is attributed to the adsorption potential of biochar and enhanced phytoremediation by Act12 in sorghum. Overall, biochar stabilized the trace elements and Act12 improved the soil enzymatic activities, which in turn reduced the plant stress, leading to low antioxidant activity. BCF, TF and MEA also confirmed the role of Act12 in the translocation of PTEs in soil contaminated by mines. The higher MEA highlighted the need of adopting multiple cropping systems as a remediation technology. PCA analysis also demonstrated the influence of the agrochemical characteristics (pH, EC) and PTEs on soil and plant enzymatic activities in soil contaminated by mines in FC. Extractable Cd and MDA had a close relationship. The higher bioavailable Cd content in soil enhance ROS formation and consequently higher MDA content, posing a severe risk to plants. Further experiments on different hyperaccumulators are needed to verify the potential of Act12 and biochar, which will provide new insight into the rehabilitation and restoration of soil contaminated by mines.

## Material and Methods

### Samples collection

**Figure 8:**
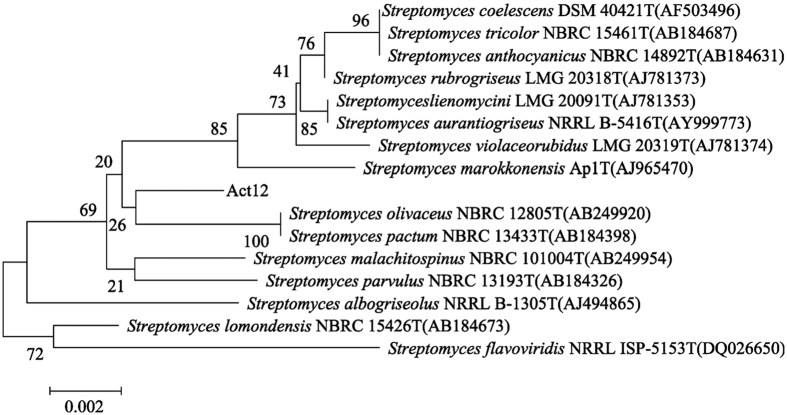
Phylogenetic tree of *Streptomyces pactum* (Act12) based on 16S rDNA.

Soil contaminated by smelters/mines was collected from Feng County (106°24′54″-107°7′30″ N, 33°34′57″-34°18′21″ E) in Shaanxi Province, China. Samples were collected from contaminated surface soil (0–20 cm), stored in polyethylene bags and transferred to the laboratory. Soil samples were homogenized, air-dried, crushed manually and passed through a 2-mm sieve. Biochar was purchased from Shaanxi Yixin Energy Company, Yangling, China. Pig manure and saw dust were mixed in a 2:1 ratio (dry weight basis) in a PVC composter (130 L) to prepare the PMC according to Li *et al*.^10^. Actinomycete (Act12) was isolated from the Qinghai-Tibet Plateau of China^24^, hereafter identified as *Streptomyces pactum*, according to its biochemical tests and 16S rDNA sequence analysis (Fig. 8). An Act12 carrier was supplied by Laboratory of Microbial Resources at College of Natural Resources and Environment, Northwest A&F University (Yangling, China). Living spore densities of preparations were 2.6 × 10^11^ spores g^−1^.

## Experimental Methods

### Pot Experiment

A pot experiment was carried out to assess the role of Act12 in the growth and PTEs phytoremediation efficiency of *Sorghum bicolor* seedlings. The treatments included a control, only 1% biochar, and different combinations of Act12 with 1% biochar. The experiment was laid out following a completely randomized design (CRD) in natural conditions under a mobile transparent shelter house at Northwest A&F University, Yangling, China. The mean temperature during the sorghum growth period ranged from 17–27.7 °C. Soil treatments were T1 (Control), T2 (1% biochar), T3 (1% biochar + 0.075% Act12), T4 (1% biochar + 0.150% Act12) and T5 (1% biochar + 0.225% Act12). Each pot with closed drainage (15 cm bottom diameter × 20 cm height × 25 cm top diameter) was filled with 2 kg of thoroughly mixed untreated/treated soil (2 mm) as growing media and 5% PMC as a nutritional supplement. Sorghum seeds were pretreated with 3% H_2_O_2_ to sterilize. Ten sorghum seeds were sown and subsequently thinned to five plants per pot, with three replicates per treatment. Tap water was frequently added to each pot to maintain 60–70% of the water holding capacity throughout the plant growth. The experiment was set up for 45 days (14 Aug–28 Sep, 2015) and the dry weight of shoots and roots were recorded after harvesting.

### Analysis of smelter soil, pig manure compost and wood biochar

Soil pH (1:2), electrical conductivity (EC) and organic matter were measured according to Li *et al*.^10^. The total nitrogen (TN), phosphorus and TOC were determined according to Wang *et al*.^11^. The soil particle size distribution (Mastersizer 2000E laser diffractometer, UK) and cation exchange capacity (CEC) were measured according to Mahar *et al*.^25^. The total trace elements in FC soil, wood biochar and PMC were measured by ICP-AES^33^. Soil DTPA/TEA extractable trace elements (Zn, Pb, Cd and Cu) were tested according to Chen *et al*.^66^. All chemical solutions were prepared using DI water, and all the chemicals were of analytical grade. After sorghum harvesting, β-glucosidase^67^, alkaline phosphatase^5^ and urease^33^ were determined in triplicate.

### Plant analysis

The chlorophyll in sorghum leaves was measured with a portable Minolta chlorophyll detector (SPAD-502, Osaka 590–8551, Japan) according to Mahar *et al*.^25^. The chlorophyll content was detected three times, and the mean SPAD values per treatment are presented. After 45 days, the shoot and root samples of sorghum were harvested, thoroughly washed with tap water followed by DI water and dried to constant weight at 105 °C. Plant dry biomass was crushed into a fine powder and stored. Shoot and root samples were digested with HNO_3_–HClO_4_ (3:1), and the total concentrations of Zn, Pb, Cd and Cu were determined^66^. The peroxidase (POD) activity was determined with guaiacol according to Lagrimini^68^. PAL and PPO activities were determined by the standard method^69^. The MDA content was measured according to Goswami and Das^48^.

### Phytoextraction indices

The amount of TEs translocated from soil to shoot and root was calculated using the following equations^62^,^63^,^70^.


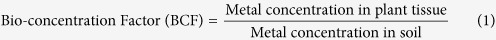



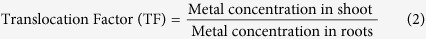



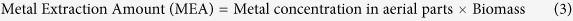


### Quality control and statistical analysis

The experiment was conducted in triplicates. Reagent blanks were used to correct the analytical values. Standard reference materials of wheat (GBW10011, National Research Center of Certified Reference Materials, Beijing, China) and soil (GBW07405, Chinese Academy of Geological Sciences) were used for quality control throughout the experimental analysis. The recovery of the standard wheat sample ranged from 91.0–100.2%, 96.7–106%, 97.9–103% and 95.4–104.4% for Cd, Cu, Pb and Zn, respectively. The recovery of the standard soil sample ranged from 90.5–102.2%, 95.8–105.8%, 94.5–104.5% and 93.6–106.2% for Cd, Cu, Pb and Zn, respectively. For the standard wheat and soil samples, the results were within the acceptable range. All the experimental data were subjected to one-way ANOVA (*p* < 0.05) for independent variable analysis and principal component analysis (PCA) using IBM SPSS Statistics 22.0 for Windows. All graphs were drawn using Origin-Pro (version 7.5).

## Additional Information

**How to cite this article**: Ali, A. *et al. Streptomyces pactum* assisted phytoremediation in Zn/Pb smelter contaminated soil of Feng County and its impact on enzymatic activities. *Sci. Rep.* **7**, 46087; doi: 10.1038/srep46087 (2017).

**Publisher's note:** Springer Nature remains neutral with regard to jurisdictional claims in published maps and institutional affiliations.

## Figures and Tables

**Table 1:** Physicochemical properties of Feng County soil, pig manure compost and biochar.

Basic Characteristics	Feng County Soil	Pig Manure Compost	Wood Biochar
pH	7.72	7.17	8.49
EC (μS cm^−1^)	422	5313	1934
Clay %	1.56	—	—
Silt %	48.43	—	—
Sand %	50.01	—	—
Soil texture	Sandy loam	—	—
CEC (cmol^+^ kg^−1^)	96.5	—	—
Organic matter (g kg^−1^)	14.9	695.4	—
Total Nitrogen (g kg^−1^)	1.23	25.83	4.51
Total Phosphorus (g kg^−1^)	0.848	26.5	0.23
Total potassium (g kg^−1^)	5.51	7.45	—
Total organic carbon (g kg^−1^)	8.64	40.3	—
**Total PTEs in FC soil, pig manure compost and biochar samples (mg kg^−1^)**			
Zn	6625	1008	115.4
Pb	204.4	15.7	25.0
Cd	117.7	0.67	0.91
Cu	51.1	675	12.6
Al	31629	—	—
As	11.1	—	—
Ca	15538	—	—
Co	16.20	—	—
Cr	61.75	—	—
Fe	25694	—	3523
Hg	0.30	—	—
Mg	8205	—	—
Mn	729.7	—	—
Mo	0.85	—	—
Na	9200	—	—
**DTPA extractable PTEs in FC soil, pig manure compost and biochar samples (mg kg^−1^)**			
Zn	584	296	—
Pb	40.2	5.32	—
Cd	36.4	0.34	—
Cu	1.77	229	—

*Values indicate mean of one sample with three replications.

**Table 2:** Bioconcentration Factor, Translocation Factor and Metal Extraction Amount of *Sorghum bicolor* after Act12 inoculation.

Trts	BCF				TF				MEA (μg plant^−1^)			
	Zn	Pb	Cd	Cu	Zn	Pb	Cd	Cu	Zn	Pb	Cd	Cu
T1	1.14	0.75	1.85	2.30	0.016	0.58	2.20	0.05	1140	51.34	114.6	6.94
T2	1.34	0.62	1.52	2.72	0.023	0.44	1.94	0.07	1374	43.60	96.67	8.40
T3	0.81	0.75	0.97	3.54	0.021	0.77	1.16	0.09	862.8	54.94	64.38	11.41
T4	1.19	0.88	1.65	3.54	0.025	0.74	2.50	0.07	1457	73.83	125.4	13.08
T5	1.22	1.27	1.85	5.19	0.023	0.84	2.44	0.08	1447	103.6	136.8	18.66

*Values indicate mean of one sample with three replications.
